# Acute-phase clinical phenotyping stratifies long-term cognitive and functional outcomes in cerebral venous sinus thrombosis

**DOI:** 10.3389/fneur.2026.1754770

**Published:** 2026-05-01

**Authors:** Wentao Lai, Jing Hu

**Affiliations:** 1 Department of Neurosurgery, Ganzhou Hospital-Nanfang Hospital, Southern Medical University, Ganzhou, China; 2 Department of Neurosurgery, Ganzhou People’s Hospital, Ganzhou, China; 3 Department of Hematology, Ganzhou Hospital-Nanfang Hospital, Southern Medical University, Ganzhou, China; 4 Department of Hematology, Ganzhou People’s Hospital, Ganzhou, China; 5 Jiangxi Health Commission Key Laboratory of Leukemia, the Affiliated Ganzhou Hospital, Jiangxi Medical College, Ganzhou, China

**Keywords:** cerebral venous sinus thrombosis, clinical phenotyping, cognitive impairment, outcome assessment, prognosis

## Abstract

**Background:**

Current prognostic models for cerebral venous sinus thrombosis (CVST) inadequately predict long-term cognitive and functional outcomes, focusing primarily on survival. We aimed to develop and validate a simple, acute-phase clinical phenotyping system to stratify the risk of these disabling sequelae.

**Methods:**

In this retrospective cohort study, consecutive adult CVST patients between 2014 and 2024 were included. Patients were classified into three acute-phase phenotypes: Benign Intracranial Hypertension, Focal Brain Injury, and Diffuse Brain Injury. Long-term outcomes (cognitive impairment, depressive symptoms, return to work) were assessed at a median of 1 year post-onset via telephone interview. Multivariable logistic regression analyses adjusted for key confounders were used to determine independent associations.

**Results:**

Among 110 patients, the prevalence of the Benign, Focal, and Diffuse Brain Injury phenotypes was 33.6% (*n* = 37), 38.2% (*n* = 42), and 28.2% (*n* = 31), respectively. A significant gradient of worsening outcomes was observed across the three phenotypes based on the dominant clinical syndrome of isolated intracranial hypertension, focal, or diffuse brain injury. The prevalence of cognitive impairment (MoCA<26) was 27.0, 59.5, and 90.3% (*p* < 0.001), and the rate of return to work was 89.2, 66.7, and 32.3% (*p* = 0.002), respectively. After adjustment, both the Focal (aOR = 3.26, 95% CI: 1.18–9.01) and Diffuse Brain Injury (aOR = 9.21, 95% CI: 2.84–29.87) phenotypes were independent predictors of cognitive impairment versus the Benign phenotype. The Diffuse Brain Injury phenotype was also independently associated with depressive symptoms (aOR = 4.85, 95% CI: 1.67–14.09) and an 85% reduced odds of returning to work (aOR = 0.15, 95% CI: 0.05–0.43).

**Conclusion:**

A simple, bedside-accessible clinical phenotyping system effectively stratifies long-term risk in CVST patients. The Diffuse Brain Injury phenotype warrants early, multidisciplinary intervention to mitigate its multifaceted poor prognosis.

## 1 Introduction

Cerebral venous sinus thrombosis (CVST) is a distinct cerebrovascular disease with highly heterogeneous clinical presentations and outcomes (1). While acute mortality has significantly decreased owing to improved diagnosis and management, a growing body of evidence indicates that a substantial proportion of survivors suffer from long-term cognitive impairment, depression, and functional disability (1–4). However, current prognostic models primarily focus on survival and gross neurological recovery, leaving the long-term cognitive sequelae (a critical determinant of quality of life) poorly characterized and predicted.

Existing attempts to understand this heterogeneity have been limited. Prognostic assessments often rely on singular indicators such as coma at onset or radiographic findings, which lack the granularity to capture the spectrum of clinical phenotypes (5–7). Consequently, there is a critical gap in a validated, clinically applicable framework that can stratify CVST patients in the acute phase according to their risk of specific long-term functional and cognitive deficits. This absence hinders the development of targeted follow-up and rehabilitation strategies.

This study aimed to investigate the association between acute-phase clinical phenotypes and long-term outcomes, and to validate these phenotypes as independent predictors after adjustment for key confounders.

## 2 Materials and methods

### 2.1 Study design and patient population

This retrospective cohort study was conducted at Ganzhou People’s Hospital. We screened all consecutive adult patients (aged ≥18 years) discharged with a diagnosis of CVST between January 1, 2014, and June 30, 2024.

The diagnosis of CVST was confirmed by either: (1) a combination of magnetic resonance imaging and magnetic resonance venography (MRI + MRV), or (2) digital subtraction angiography (DSA), in accordance with the European Federation of Neurological Societies guidelines (8).

**Figure 1:**
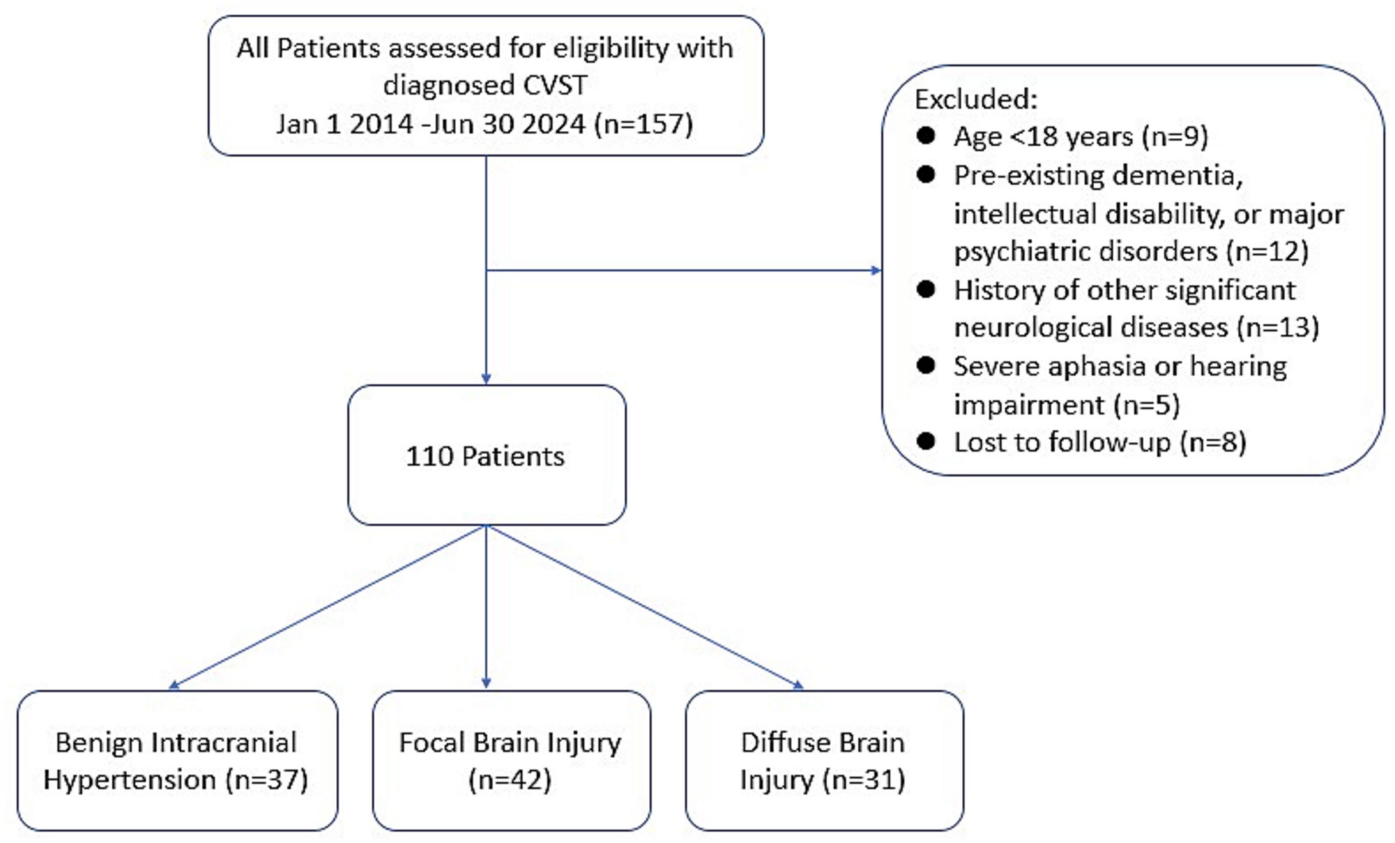
Flow diagram of patient enrollment.

Patients were included if they survived until the last follow-up and the interval from the acute event to the final assessment was at least 1 year, ensuring evaluation in the chronic recovery phase. Key exclusion criteria were: (1) pre-existing dementia, intellectual disability, or major psychiatric disorders; (2) history of other significant neurological diseases (e.g., brain tumor, prior large-scale stroke) that could confound cognitive assessment; (3) severe aphasia or hearing impairment precluding reliable telephone interview; and (4) insufficient clinical data for accurate phenotyping. The study was approved by the Institutional Review Board of Ganzhou People’s Hospital (Approval No.: 2024081116), and informed consent was obtained from all participants. Given the rarity of CVST, we included all eligible consecutive patients during the study period to maximize sample size, in lieu of a formal sample size calculation. The flow of patient enrollment is summarized in Figure 1.

### 2.2 Data extraction and acute-phase clinical phenotyping

Baseline data were systematically extracted from electronic medical records by two trained neurologists blinded to the long-term outcomes. Collected variables included:

Demographics: age, sex, and years of formal education.

Acute Disease Characteristics: admission Glasgow Coma Scale (GCS) score, presence of coma, and acute symptomatic seizures.

Radiologic Features: involvement of the venous sinus system (e.g., superior sagittal sinus, transverse sinus, sigmoid sinus, and rectus sinus) and presence of parenchymal lesions (hemorrhagic infarction or edema).

Etiology and Risk Factors: thrombophilia, recent infection(head and neck infection, systemic infection, other localized infection), dehydration, and oral contraceptive use.

Treatment: Treatment variables extracted from medical records included: Anticoagulation, Endovascular therapy, Intracranial hypertension management, Antiseizure medications. However, due to the retrospective nature of this study and incomplete documentation over the 10-year study period (2014–2024), the following variables were not reliably available for analysis:** recanalization status (follow-up imaging was not uniformly performed), detailed anticoagulation target ranges, and consistent records of intracranial pressure-directed therapies beyond initial hospitalization. These limitations are addressed in the Discussion.

Patients were classified into three mutually exclusive clinical phenotypes based on the dominant clinical presentation during the acute phase (from admission to discharge or clinical stabilization): Benign Intracranial Hypertension Type: Characterized by symptoms and signs of isolated intracranial hypertension (e.g., headache, papilledema, visual obscurations) without objective evidence of focal neurological deficits or impaired consciousness. Focal Brain Injury Type: Dominated by clinical features suggesting focal cortical or subcortical dysfunction, including focal neurological deficits (e.g., hemiparesis, aphasia) or seizure activity. Diffuse Brain Injury Type: Characterized by evidence of widespread cerebral dysfunction, such as encephalopathy, delirium, or multifocal signs, typically reflecting the most severe end of the clinical spectrum.

The phenotyping was performed independently by two neurologists who were blinded to the long-term outcome data. Any discrepancies were resolved through discussion with a third senior researcher. The inter-rater reliability for phenotyping was excellent (Cohen’s kappa = 0.92, *p* < 0.001).

### 2.3 Long-term outcome assessment

All long-term outcomes were assessed via a single structured telephone interview conducted at a cross-sectional time point for each patient. The interview was performed at a median of 12 months (interquartile range IQR: 10–15 months; range: 9–24 months) after the acute CVST event. The specific follow-up time for each patient was calculated from the date of hospital admission to the date of the telephone interview. The assessment battery included:

(1) Global Cognition: The Telephone-Montreal Cognitive Assessment (T-MoCA) was used, with a total score of <26 defining cognitive impairment (9).
(2) Specific Cognitive Domains:

Executive Function/Attention: Assessed using the Digit Span Test (forward and backward).
Verbal Memory: Evaluated by the delayed recall component of the Hopkins Verbal Learning Test-Revised (HVLT-R).

(3) Emotional State: Depressive symptoms were screened using the 2-item Patient Health Questionnaire (PHQ-2), with a score of ≥3 indicating significant symptoms (10).
(4) Functional Outcome: Return to pre-morbid full-time work was defined as the patient’s self-report of having returned to their same or equivalent paid employment with similar working hours and responsibilities as before the CVST. Those who retired early, switched to part-time work, downgraded to a less demanding position, or transitioned to unpaid household work were classified as not having returned to pre-morbid work.

### 2.4 Statistical analysis

Data are presented as mean ± standard deviation, median [interquartile range], or number (percentage), as appropriate. Baseline characteristics and outcomes across the three phenotypes were compared using one-way ANOVA, Kruskal–Wallis test, or Pearson’s chi-square test, with *post-hoc* comparisons performed using Bonferroni correction (for ANOVA) or Dunn’s test (for Kruskal–Wallis test), as appropriate.

The primary outcome was the presence of cognitive impairment (MoCA<26). Multivariable logistic regression analysis was employed to examine the independent association between clinical phenotypes and the primary outcome, as well as the secondary outcomes (depressive symptoms and return to work). The models were adjusted for pre-specified covariates deemed clinically relevant or with *p* < 0.1 in univariate analysis: age, sex, education years, admission GCS score, presence of parenchymal lesion, and endovascular treatment. Results are reported as adjusted odds ratios (aOR) with 95% confidence intervals (CI). Model fit was assessed using the Hosmer-Lemeshow test, and the explanatory power was reported using Nagelkerke’s *R*^2^. A two-tailed *p*-value < 0.05 was considered statistically significant. All analyses were performed using SPSS Statistics (Version 26.0, IBM Corp, Armonk, NY).

## 3 Results

### 3.1 Study population and clinical phenotyping

A total of 110 CVST patients who met the inclusion criteria were included in the final analysis. Based on the acute-phase clinical presentation, patients were classified into three distinct phenotypes: Benign Intracranial Hypertension (*n* = 37, 33.6%), Focal Brain Injury (*n* = 42, 38.2%), and Diffuse Brain Injury (*n* = 31, 28.2%).

### 3.2 Baseline characteristics across clinical phenotypes

The baseline characteristics of the study population stratified by clinical phenotype are summarized in Table 1. There were no significant differences in demographic factors, including age, sex, and years of education, among the three groups (all *p* > 0.05).

**Table 1:** Baseline characteristics stratified by clinical phenotype.

Characteristic	Benign intracranial hypertension (*n* = 37)	Focal brain injury (*n* = 42)	Diffuse brain injury (*n* = 31)	Statistical value	*p*-value
Population statistics					
Age (years)	38.1 ± 12.5	42.8 ± 13.0	44.3 ± 14.1	*F*(2, 107) = 2.14	0.123
Female sex, *n* (%)	30 (81.1%)	28 (66.7%)	17 (54.8%)	*χ*^2^ = 5.43	0.066
Education (year)	6.2 ± 2.2	5.9 ± 2.4	5.1 ± 1.6	F(2, 107) = 2.36	0.100
Clinical features in the acute phase					
Admission GCS Score, median [IQR]	15 [15–15]	15 [14–15]	13 [9–14]	H = 61.5	<0.001
Coma, *n* (%)	0 (0%)	2 (4.8%)	16 (51.6%)	*χ*^2^ = 39.51	<0.001
Acute symptomatic seizure, *n* (%)	2 (5.4%)	16 (38.1%)	10 (32.3%)	*χ*^2^ = 12.13	<0.001
Radiologic characteristic					
Involvement of the deep venous system, *n* (%)	2 (5.4%)	5 (11.9%)	12 (38.7%)	*χ*^2^ = 14.46	<0.001
Parenchymal lesion (hemorrhage or infarction), *n* (%)	5 (13.5%)	35 (83.3%)	19 (61.3%)	*χ*^2^ = 39.58	<0.001
Etiology and risk factors					
Oral contraceptives (for women), n/*n* (%)	2/30 (6.7%)	2/28 (7.1%)	1/17 (5.9%)	*χ*^2^ = 0.03	0.987
Thrombophilia, *n* (%)	9 (24.3%)	10 (23.8%)	6 (19.4%)	*χ*^2^ = 0.28	0.87
Recent infection, *n* (%)	5 (13.5%)	5 (11.9%)	4 (12.9%)	*χ*^2^ = 0.05	0.98
Head and neck infection	3 (8.1%)	4 (9.5%)	2 (6.5%)	*χ*^2^ = 0.22	0.89
Systemic infection	2 (5.4%)	1 (2.4%)	2 (6.5%)	*χ*^2^ = 0.78	0.68
Treatment					
Endovascular treatment, *n* (%)	3 (8.1%)	8 (19.0%)	13(41.9%)	*χ*^2^ = 9.09	0.011
Comorbidities					
Hypertension, *n* (%)	8 (21.6%)	12 (28.6%)	9 (29.0%)	*χ*^2^ = 0.71	0.70
Diabetes mellitus, *n* (%)	3 (8.1%)	5 (11.9%)	4 (12.9%)	*χ*^2^ = 0.55	0.76
Hyperlipidemia, *n* (%)	6 (16.2%)	8 (19.0%)	6 (19.4%)	*χ*^2^ = 0.15	0.93

Data are presented as mean ± standard deviation, median [interquartile range], or *n* (%). *p*-values were derived from one-way ANOVA, Kruskal–Wallis test, or Pearson’s chi-square test as appropriate. *Post-hoc* analyses were performed with Bonferroni correction.

However, marked differences were observed in acute-phase disease severity and radiological findings. Patients with the Diffuse Brain Injury phenotype had significantly lower admission GCS scores (median 13 [IQR 9–14]) and a substantially higher frequency of coma (51.6%) compared to the other groups (*p* < 0.001 for both). Acute symptomatic seizures was also more common in the Focal (38.1%) and Diffuse (32.3%) Brain Injury groups compared to the Benign group (5.4%, *p* < 0.001). Radiologically, involvement of the deep venous system and parenchymal lesions (hemorrhage or infarction) were significantly more prevalent in the Diffuse Brain Injury phenotype (38.7 and 61.3%, respectively), followed by the Focal Brain Injury group (*p* < 0.001 for both). Consequently, the rate of endovascular treatment was highest in the Diffuse Brain Injury group (41.9%, *p* = 0.011). Etiology and risk factors were comparable across all phenotypes.

Regarding etiology, a recent infection within 4 weeks prior to onset was identified in 14 patients (12.7% of the total cohort). Among these, 9 (64.3%) were head and neck infections (sinusitis in 4, otitis media in 3, dental infection in 2) and 5 (35.7%) were systemic infections (pneumonia in 3, urinary tract infection in 2). Dehydration was documented in 4 patients (3.6%). Notably, no identifiable etiological factor was found in 62 patients (56.4% of the cohort), who were classified as idiopathic CVST. The distribution of etiologies did not differ significantly across the three clinical phenotypes (all *p* > 0.05; Table 1).

### 3.3 Long-term cognitive and functional outcomes

The long-term outcomes assessed at a median of 1 year post-onset are presented in Table 2. A significant gradient of worsening outcomes across the clinical phenotypes was evident for all measured domains.

**Table 2:** Long-term outcomes stratified by clinical phenotype.

Cognitive function indicators	Benign intracranial hypertension (*n* = 37)	Focal brain injury (*n* = 42)	Diffuse brain injury (*n* = 31)	Statistical value	*p*-value
Overall cognitive function					
MoCA	26.2 ± 2.1	23.5 ± 3.2	18.9 ± 4.8	*F*(2, 107) = 38.30	<0.001
Cognitive impairment (MoCA<26), *n* (%)	10 (27.0%)	25 (59.5%)	28 (90.3%)	*χ*^2^ = 26.32	<0.001
Specific cognitive domain					
Executive function/attention (digital breadth)	10.5 ± 2.2	9.8 ± 2.5	6.8 ± 2.7	*F*(2, 107) = 21.11	<0.001
Verbal memory (HVLT-R delayed recall)	8.8 ± 2.5	7.5 ± 2.8	4.5 ± 2.9	*F*(2, 107) = 21.65	<0.001
Emotional state					
Depressive symptoms (PHQ-2 ≥ 3), *n* (%)	8 (21.6%)	15 (35.7%)	20 (64.5%)	*χ*^2^ = 10.08	0.006
Functional outcome					
Return to full-time work, *n* (%)	33 (89.2%)	28 (66.7%)	10 (32.3%)	*χ*^2^ = 12.34	0.002

MoCA, Montreal Cognitive Assessment; HVLT-R, Hopkins Word Learning Test–Revised Edition; PHQ-2, Patient Health Questionnaire −2 items.

Data are presented as mean ± standard deviation or n (%). *p*-values were derived from one-way ANOVA for continuous variables and Pearson’s chi-square test for categorical variables.

For global cognitive function, the MoCA total score progressively declined from the Benign (26.2 ± 2.1) to the Focal (23.5 ± 3.2) and the Diffuse Brain Injury (18.9 ± 4.8) groups (*p* < 0.001). Accordingly, the prevalence of cognitive impairment (MoCA<26) was 27.0, 59.5, and 90.3% in the three groups, respectively (*p* < 0.001). This gradient was also observed in specific cognitive domains, including executive function/attention and verbal memory (p < 0.001 for both).

In the Diffuse Brain Injury phenotype, 80.6% (25/31) of patients scored below the normative threshold for executive function (Digit Span forward < 8), and 74.2% (23/31) showed impaired verbal memory (HVLT-R delayed recall < 5). In contrast, impairment rates in the Benign phenotype were 18.9 and 13.5%, respectively.

Furthermore, the prevalence of depressive symptoms (PHQ-2 ≥ 3) was highest in the Diffuse Brain Injury group (64.5%), compared to the Focal (35.7%) and Benign (21.6%) groups (*p* = 0.006). Finally, the rate of return to full-time work demonstrated a stark contrast, with 89.2% of the Benign group returning to work, versus 66.7% in the Focal and only 32.3% in the Diffuse Brain Injury group (*p* = 0.002).

### 3.4 Multivariable regression analyses

To determine the independent association of clinical phenotypes with long-term outcomes, multivariable logistic regression analyses were performed, adjusting for age, sex, education, admission GCS score, parenchymal lesion, and endovascular treatment.

As shown in Table 3, both the Focal Brain Injury (aOR = 3.26, 95% CI: 1.18–9.01, *p* = 0.023) and Diffuse Brain Injury (aOR = 9.21, 95% CI: 2.84–29.87, *p* < 0.001) phenotypes were independent predictors of cognitive impairment compared to the Benign phenotype. Higher age (aOR = 1.04, *p* = 0.048) and the presence of a parenchymal lesion (aOR = 3.49, *p* = 0.021) were also independently associated with cognitive impairment, whereas higher education (aOR = 0.84, *p* = 0.031) and admission GCS score (aOR = 0.84, *p* = 0.042) were protective factors. The model demonstrated good fit (Hosmer-Lemeshow *p* = 0.561).

**Table 3:** Multivariate logistic regression analysis of factors associated with cognitive impairment (MoCA < 26) in CVST patients.

Variable	*β*	*SE*	Adjusted OR (95% CI)	*p*-value
Clinical phenotype				
Benign intracranial hypertension	Ref	–	1.00	–
Focal brain injury	1.18	0.52	3.26 (1.18–9.01)	0.023
Diffuse brain injury	2.22	0.60	9.21 (2.84–29.87)	<0.001
Age, per 1-year increase	0.04	0.02	1.04 (1.00–1.09)	0.048
Female Sex	0.38	0.47	1.46 (0.58–3.68)	0.421
Education (per year)	−0.17	0.08	0.84 (0.72–0.99)	0.031
Admission GCS score	−0.18	0.09	0.84 (0.71–0.99)	0.042
Parenchymal lesion	1.25	0.54	3.49 (1.21–10.08)	0.021
Endovascular treatment	0.78	0.49	2.18 (0.84–5.68)	0.112

Model fit statistics: Hosmer–Lemeshow test: *χ*^2^ = 6.78, *P* = 0.561. Nagelkerke *R*^2^ = 0.447.

Table 4 presents the analysis for depressive symptoms. The Diffuse Brain Injury phenotype remained a strong independent predictor (aOR = 4.85, 95% CI: 1.67–14.09, *p* = 0.004). The association for the Focal Brain Injury phenotype was not statistically significant (aOR = 2.27, *p* = 0.095).

**Table 4:** Multivariate logistic regression analysis of factors associated with depressive symptoms (PHQ-2 ≥ 3) in CVST patients.

Variable	*β*	*SE*	Adjusted OR (95% CI)	*p*-value
Clinical phenotype				
Benign intracranial hypertension	Ref	–	1.00	–
Focal brain injury	0.82	0.47	2.27 (0.87–5.93)	0.095
Diffuse brain injury	1.58	0.51	4.85 (1.67–14.09)	0.004
Age, per 1-year increase	0.02	0.02	1.02 (0.98–1.06)	0.317
Female sex	0.35	0.44	1.42(0.60–3.38)	0.426
Education (per year)	−0.09	0.08	0.91 (0.78–1.07)	0.260
Admission GCS score	−0.11	0.10	0.90 (0.74–1.09)	0.268
Parenchymal lesion	0.58	0.48	1.79 (0.68–4.70)	0.245
Endovascular treatment	0.55	0.49	1.73 (0.66–4.55)	0.265

Model fit statistics: Hosmer–Lemeshow test: *χ*^2^ = 6.85, *p* = 0.553. Nagelkerke *R*^2^ = 0.265.

The Diffuse Brain Injury phenotype corresponds to an 85% reduction in the odds of returning to work compared to the Benign phenotype (aOR = 0.15, 95% CI: 0.05–0.43, *p* = 0.001, Table 5). A higher admission GCS score was a significant protective factor for returning to work (aOR = 1.38, *p* = 0.008).

**Table 5:** Multivariate logistic regression analysis of factors associated with return to full-time work in CVST patients.

Variable	*β*	*SE*	Adjusted OR (95% CI)	*p*-value
Clinical phenotype				
Benign intracranial hypertension	Ref	–	1.00	–
Focal brain injury	−0.95	0.51	0.39 (0.13–1.15)	0.088
Diffuse brain injury	−1.92	0.55	0.15 (0.05–0.43)	0.001
Age, per 1-year increase	−0.03	0.02	0.97 (0.93–1.01)	0.134
Female sex	−0.45	0.47	0.64 (0.25–1.62)	0.340
Education (per year)	0.16	0.08	1.17 (1.00–1.38)	0.061
Admission GCS score	0.32	0.11	1.38 (1.09–1.74)	0.008
Parenchymal lesion	−0.85	0.50	0.43 (0.16–1.15)	0.089
Endovascular treatment	−0.62	0.51	0.54 (0.20–1.47)	0.263

Model fit statistics: Hosmer–Lemeshow test: *χ*^2^ = 5.68, *p* = 0.683. Nagelkerke *R*^2^ = 0.423.

## 4 Discussion

The principal finding of this retrospective cohort study is that a simple, clinically based acute-phase phenotyping system powerfully stratifies CVST patients according to their risk of long-term cognitive impairment, depressive symptoms, and functional disability. Our results demonstrate that the Diffuse Brain Injury phenotype serves as a independent predictor of a comprehensively unfavorable long-term outcome, even after rigorous adjustment for key demographic, clinical, and radiological confounders.

The primary innovation of our study lies in proposing a holistic phenotypic classification that synthesizes multiple clinical features into clinically coherent profiles, moving beyond singular prognostic markers. This framework effectively translates a combination of clinical and radiological severities into a clinically actionable prognostic tool. While previous studies have identified isolated factors such as coma, cerebral hemorrhage, or deep venous system thrombosis as poor prognostic indicators (11), these factors often coexist and their collective impact on long-term cognitive and functional recovery remains nebulous. Our tripartite classification synthesizes these elements into clinically coherent profiles. The gradient of worsening outcomes—from Benign to Focal to Diffuse—across all measured domains (cognition, mood, and function) validates the construct validity and prognostic utility of this framework. This provides a paradigm shift from asking “Is this patient at risk?” to a more nuanced question: “What is the specific spectrum of long-term challenges this patient is likely to face?”

The profoundly poor outcomes associated with the Diffuse Brain Injury phenotype are multifactorial. Clinically, it signifies widespread cerebral dysfunction (e.g., encephalopathy, coma), which is corroborated radiologically by its strong association with deep venous system involvement and parenchymal lesions—markers of an extensive thrombotic burden and tissue injury (12). Pathophysiologically, this pattern of injury likely disrupts global cerebral networks and subcortical structures crucial for attention, arousal, and executive function (13–16), explaining the pervasive deficits observed. In contrast, the Focal Brain Injury phenotype, while still conferring significant risk for cognitive impairment, may result in more circumscribed deficits aligned with the location of the focal lesion, explaining its intermediate prognostic position (17).

Furthermore, our findings extend beyond the validation of the phenotyping system itself to offer a more nuanced understanding of CVST prognosis. The hierarchical risk gradient, from Benign to Focal to Diffuse, for a spectrum of outcomes including global cognition, executive function, verbal memory, mood, and vocational capacity, suggests that these phenotypes capture fundamental differences in the underlying brain injury. The Benign phenotype, largely sparing the parenchyma, primarily manifests reversible hypertension, resulting in minimal long-term sequelae. The Focal phenotype, often associated with localized venous infarcts or hemorrhages, leads to deficits contingent upon the site of injury, which can be disabling but not necessarily pervasive. In stark contrast, the Diffuse Brain Injury phenotype, strongly linked to deep venous system involvement and parenchymal lesions, likely signifies a more profound and widespread pathophysiological state. The deep venous system drains critical subcortical and deep white matter structures involved in neural integration, arousal, and mood regulation (18–20). Its thrombosis may disrupt global cerebral networks and subcortical circuits, providing a plausible anatomical and functional basis for the observed triad of severe executive dysfunction, memory impairment, and high depression rates, a combination that profoundly impedes functional recovery and return to work (21, 22). Thus, our phenotypic classification effectively translates a combination of clinical and radiological severities into a clinically actionable prognostic tool that reflects the anatomical extent and functional disruption of the thrombotic event. While imaging features (deep system involvement, parenchymal lesions) are strongly associated with the Diffuse phenotype, our phenotyping system is designed to be used at bedside based on clinical presentation alone. Imaging can serve as a confirmatory or supplementary tool, particularly in equivocal cases.

Our findings align with and extend the existing body of knowledge on long-term CVT outcomes. The landmark International Study on Cerebral Vein and Dural Sinus Thrombosis (ISCVT) established that 6-month functional independence (mRS 0–1) is achieved in approximately 79% of patients, but also highlighted that deep venous system involvement and parenchymal lesions are strong predictors of death or dependency (23). Our proposed clinical phenotyping system translates these established radiographic and clinical risk factors into a bedside-accessible, holistic prognostic profile. Specifically, the markedly poor outcomes in our Diffuse Brain Injury group (90.3% cognitive impairment) provide a mechanistic explanation for the ISCVT findings, attributing the disability not merely to motor deficits but to a profound disruption of global cognitive networks and executive function.

Furthermore, the granular long-term outcomes reported by Hiltunen et al. revealed that despite favorable mRS scores, up to 44% of CVST survivors fail to return to their previous vocational status, and over half experience residual cognitive fatigue (4). While Heldner et al. developed a clinical score to predict acute CVST diagnosis using D-dimer levels, our work focuses on post-acute prognostic stratification using bedside clinical phenotypes (24). Our study corroborates this “hidden burden” of CVST. By stratifying this risk, we demonstrate that the return-to-work rate plummets to 32.3% in the Diffuse phenotype compared to 89.2% in the Benign phenotype. This underscores that the Benign Intracranial Hypertension group largely drives the favorable mRS statistics in previous cohort studies, while the Focal and Diffuse groups shoulder the majority of the long-term neuropsychiatric morbidity described by Hiltunen and colleagues. The 2024 American Heart Association (AHA) Scientific Statement emphasizes the need for multidisciplinary follow-up and acknowledges the limitations of relying solely on mRS for outcome assessment in CVT (25). The ESO guidelines similarly recommend vigilance for late seizures and cognitive sequelae (26). By demonstrating that the Diffuse Brain Injury phenotype predicts a five-fold increase in depressive symptoms and an 85% reduction in return-to-work odds. It enables clinicians to transition from reactive management of complications to proactive, phenotype-specific allocation of cognitive rehabilitation and mental health resources, as recently advocated by Sánchez van Kammen et al. in their analysis of late seizures and long-term morbidity (27).

Our findings have direct clinical implications. The identification of a Diffuse Brain Injury phenotype at admission should trigger alerts for a high-risk trajectory. This necessitates not only aggressive acute management but also the early initiation of a structured, multidisciplinary long-term follow-up plan encompassing cognitive rehabilitation, psychiatric screening, and vocational support. Conversely, the relatively favorable outcomes in the Benign Intracranial Hypertension group suggest that resources can be more efficiently allocated, with follow-up focused on monitoring for headache and visual outcomes rather than pervasive cognitive decline.

Our study has several limitations that should be considered when interpreting the results. First, its retrospective and single-center design may introduce selection bias and limit the generalizability of our findings. Second, the extended study period (2014–2024), while enabling the accumulation of a substantial cohort for a rare disease like CVST, inherently spans eras of potential evolution in diagnostic sensitivities, acute treatment protocols, and rehabilitation practices. While we adjusted for endovascular therapy, we could not reliably capture several other treatments that may influence long-term outcomes, including: (1) the intensity and duration of anticoagulation (e.g., time in therapeutic range for warfarin), (2) recanalization status assessed by follow-up imaging (not routinely performed), (3) specific interventions for intracranial hypertension (e.g., serial lumbar punctures, shunting), and (4) consistent use of antiseizure medications beyond the acute phase. These variables were either incompletely documented in electronic medical records over the 10-year study period or subject to individual clinician preference. The absence of these data means we cannot rule out residual confounding. These unmeasured confounders may influence long-term outcomes, and our findings should be interpreted with this limitation. While telephone-based cognitive assessment enhanced follow-up rates, it may lack the sensitivity of comprehensive in-person neuropsychological batteries. Our outcome assessment was limited to a single cross-sectional time point at approximately 1 year post-onset. Consequently, we cannot evaluate the trajectory of cognitive recovery or the potential for late-onset depression beyond this time point. Longitudinal studies with serial assessments are needed to characterize the natural history of these outcomes. Finally, unmeasured confounders, such as cognitive reserve, social support, and the intensity of post-discharge rehabilitation, could have influenced the outcomes. Return to work is also influenced by social determinants (job type, workplace accommodations, urban–rural disparities) not captured in our clinical dataset. Our findings should be interpreted as the clinical contribution to this multifactorial outcome. Furthermore, the PHQ-2 was used solely as a screening tool for depressive symptoms; positive screens (score ≥3) were not systematically followed up with a diagnostic interview (e.g., PHQ-9) or confirmed mental health referral due to the retrospective nature of the study. This may overestimate the prevalence of clinically significant depression and is acknowledged as a limitation.

Based on our findings, we propose an initial, evidence-informed framework for phenotype-specific management. Diffuse Brain Injury Phenotype: Mandatory screening for cognitive impairment (using MoCA) and depression (PHQ-9) at 1–3 months post-discharge. Proactive referral to cognitive rehabilitation, mental health services, and vocational rehabilitation programs. Focal Brain Injury Phenotype: Targeted neuropsychological assessment based on lesion location (e.g., language, motor, or domain-specific memory). Seizure management and monitoring for focal deficits. Benign Intracranial Hypertension Phenotype: Emphasis on monitoring visual outcomes (formal perimetry if papilledema persists) and headache management. Routine cognitive screening may have lower yield.

## 5 Conclusion

In this cohort study, we developed and validated a simple, bedside-accessible clinical phenotyping system for the acute phase of CVST. This classification stratified patients into distinct prognostic trajectories for long-term cognitive impairment, depressive symptoms, and functional recovery. The Diffuse Brain Injury phenotype was identified as a potent, independent predictor of a comprehensively unfavorable outcome, underscoring its clinical utility as a key risk indicator. Our findings advocate for the integration of this phenotypic stratification into routine clinical practice to facilitate early identification of high-risk individuals, thereby informing personalized follow-up and rehabilitation pathways. Future prospective, multicenter studies are needed to externally validate these phenotypes and to investigate the efficacy of phenotype-specific management strategies.

## Data Availability

The raw data supporting the conclusions of this article will be made available by the authors, without undue reservation.
